# The frequency of yeast [*PSI*^+^] prion formation is increased during chronological ageing

**DOI:** 10.15698/mic2017.04.568

**Published:** 2017-03-27

**Authors:** Shaun H. Speldewinde, Chris M. Grant

**Affiliations:** 1 University of Manchester, Faculty of Biology, Medicine and Health, The Michael Smith Building, Oxford Road, Manchester, M13 9PT, UK.

**Keywords:** prion, yeast, chronological ageing, autophagy

## Abstract

Ageing involves a time-dependent decline in a variety of intracellular mechanisms and is associated with cellular senescence. This can be exacerbated by prion diseases which can occur in a sporadic manner, predominantly during the later stages of life. Prions are infectious, self-templating proteins responsible for several neurodegenerative diseases in mammals and several prion-forming proteins have been found in yeast. We show here that the frequency of formation of the yeast [*PSI^+^*] prion, which is the altered form of the Sup35 translation termination factor, is increased during chronological ageing. This increase is exacerbated in an *atg1 *mutant suggesting that autophagy normally acts to suppress age-related prion formation. We further show that cells which have switched to [*PSI^+^*] have improved viability during chronological ageing which requires active autophagy. [*PSI^+^*] stains show increased autophagic flux which correlates with increased viability and decreased levels of cellular protein aggregation. Taken together, our data indicate that the frequency of [*PSI^+^*] prion formation increases during yeast chronological ageing, and switching to the [*PSI^+^*] form can exert beneficial effects via the promotion of autophagic flux.

## INTRODUCTION

Biological ageing involves a progressive decline in the ability of an organism to survive stress and disease. It is a complex process which is influenced by both genetic and environmental factors 1. Common features of ageing include decreased resistance to stress, increased rates of apoptosis, a decline in autophagy and an elevated accumulation of protein aggregates 2. In humans, ageing correlates with an increased frequency of age-related diseases including heart disease, metabolic syndromes and neurodegenerative diseases such as Alzheimer’s, Parkinson’s and dementia 3.

Prions are aberrant, infectious protein conformations which can self-replicate 4. They are causally responsible for transmissible spongiform encephalopathies (TSEs) that cause several incurable neurodegenerative diseases in mammals 5. The underlying cause of TSEs is the structural conversion of a soluble prion protein (PrP^C^) into a prion form (PrP^sc^) that is amyloidogenic. The amyloid form of the protein can subsequently convert other soluble molecules into the prion form thus resulting in the accumulation of the aberrant proteins in neuronal cells 6,7. Human prion diseases are predominantly sporadic constituting approximately 70% of all cases with higher frequencies occurring during advanced age 8. There are several prion-forming proteins in yeast with the best-characterized being [*PSI*^+^] and [*PIN*^+^], which are formed from the Sup35 and Rnq1 proteins, respectively 9,10. [*PSI*^+^] is the altered conformation of the Sup35 protein, which normally functions as a translation termination factor during protein synthesis. The *de novo* formation of [*PSI*^+^] is enhanced by the presence of the [*PIN*^+^] prion, which is the altered form of the Rnq1 protein whose native protein function is unknown 11.

We have previously shown that autophagy protects against Sup35 aggregation and *de novo *[*PSI^+^*] prion formation 12. Autophagy is an intracellular quality control pathway that degrades damaged organelles and protein aggregates via vacuolar/lysosomal degradation 13. It proceeds in a highly sequential manner leading to the formation of a double-membrane-bound vesicle called the autophagosome. Fusion of the autophagosome with vacuoles/lysosomes introduces acidic hydrolases that degrade the contained proteins and organelles. Autophagy has been implicated in the ageing process and, for example, pharmacological interventions which induce autophagy result in lifespan extension during yeast chronological ageing 14,15. Autophagy appears to play a protective role in the ageing process since dysregulated autophagy is implicated in the accumulation of abnormal proteins associated with several age-related diseases including Alzheimer’s, Parkinson’s and Huntington’s diseases 3,16,17.

Yeast cells can survive for prolonged periods of time in culture and have been used as a model of the chronological life span (CLS) of mammals, particularly for tissues composed of non-dividing populations 18. Additionally, ageing is followed by replicative lifespan, which is defined as the number of budding daughter cells that originates from a particular mother yeast cell before it reaches senescence 19,20. Given that amyloidoses are typically diseases of old-age, yeast prions might be expected to form at a higher frequency in aging yeast cells. However, a study using the yeast replicative ageing model found that ageing does not increase the frequency of prion formation 21. In this current study we have examined [*PSI*^+^] prion formation using the yeast CLS model and found that the frequency of prion formation is increased during ageing. Furthermore, this frequency is elevated in an autophagy mutant suggesting that autophagy normally acts to suppress age-dependent prion formation. We show that the prion-status of cells influences CLS in an autophagy-dependent manner suggesting that prions can be beneficial in aged populations of yeast cells.

## RESULTS

### The frequency of *de novo *[*PSI^+^*] formation increases during chronological ageing

**Figure 1:**
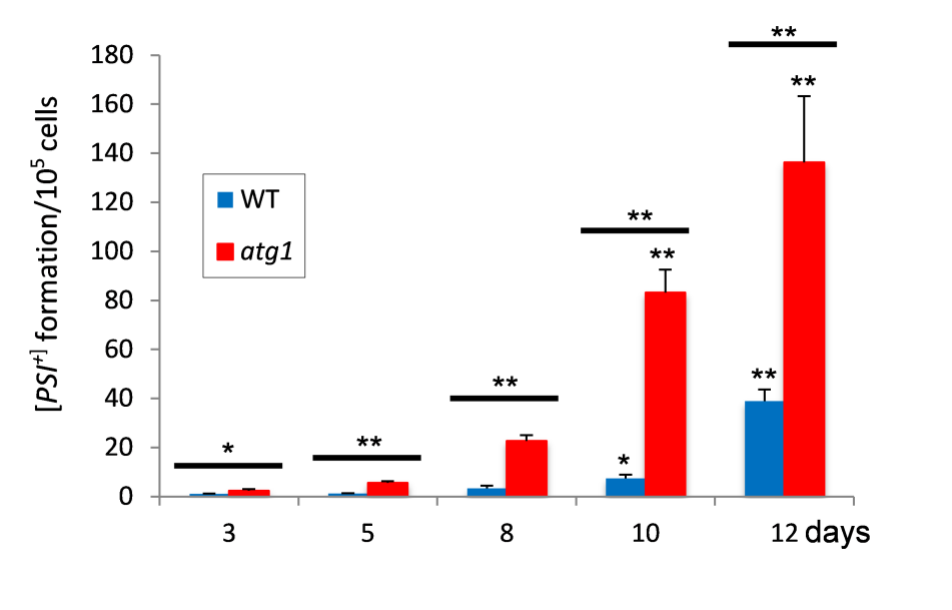
FIGURE 1: Increased frequency of [*PSI^+^*] prion formation during chronological lifespan. [*PIN^+^*][*psi^-^*] versions of the wild-type and *atg1* mutant stains were grown to stationary phase in SCD media and the frequency of [*PSI^+^*] formation measured over time. [*PSI*^+^] formation was quantified using the *ade1-14* mutant allele by growth on media lacking adenine and differentiated from nuclear mutations by their irreversible elimination in GdnHCl. Data shown are the means of three independent biological repeat experiments expressed as the number of colonies per 10^5^ viable cells. Error bars denote standard deviation. Significance is shown comparing the wild-type and *atg1 *mutant strains over time (above bars) as well as between the wild-type and *atg1 *mutant at each time-point (between bars). Statistical analysis was performed by one-way ANOVA: *p < 0.05, **p < 0.01 .

We examined yeast CLS to determine whether there is an increased frequency of [*PSI^+^*] appearance during ageing. Cultures were grown to stationary phase in liquid SCD media and prion formation measured over time. [*PSI*^+^] prion formation was quantified using the *ade1-14* mutant allele which confers adenine auxotrophy and prions differentiated from nuclear gene mutations by their irreversible elimination in guanidine hydrochloride (GdnHCl). The frequency of *de novo *[*PSI*^+^] prion formation in a control [*PIN*^+^][*psi^-^*] strain grown to stationary phase was estimated to be 1.1 x 10^-5^ comparable to previously reported frequencies 12,22. This frequency increased during CLS and a 39-fold increase was observed by day 12 (Fig. 1). We next examined the frequency of [*PSI*^+^] prion formation in an *atg1 *autophagy mutant. Atg1 is a serine/threonine kinase which is responsible for the initiation of autophagy 13,23. The frequency of [*PSI*^+^] prion formation was further elevated in the *atg1 *mutant compared with a wild-type strain, suggesting that autophagy normally acts to suppress [*PSI*^+^] prion formation during ageing (Fig. 1).

### [*PSI*^+^] increase longevity in a yeast CLS model

**Figure 2:**
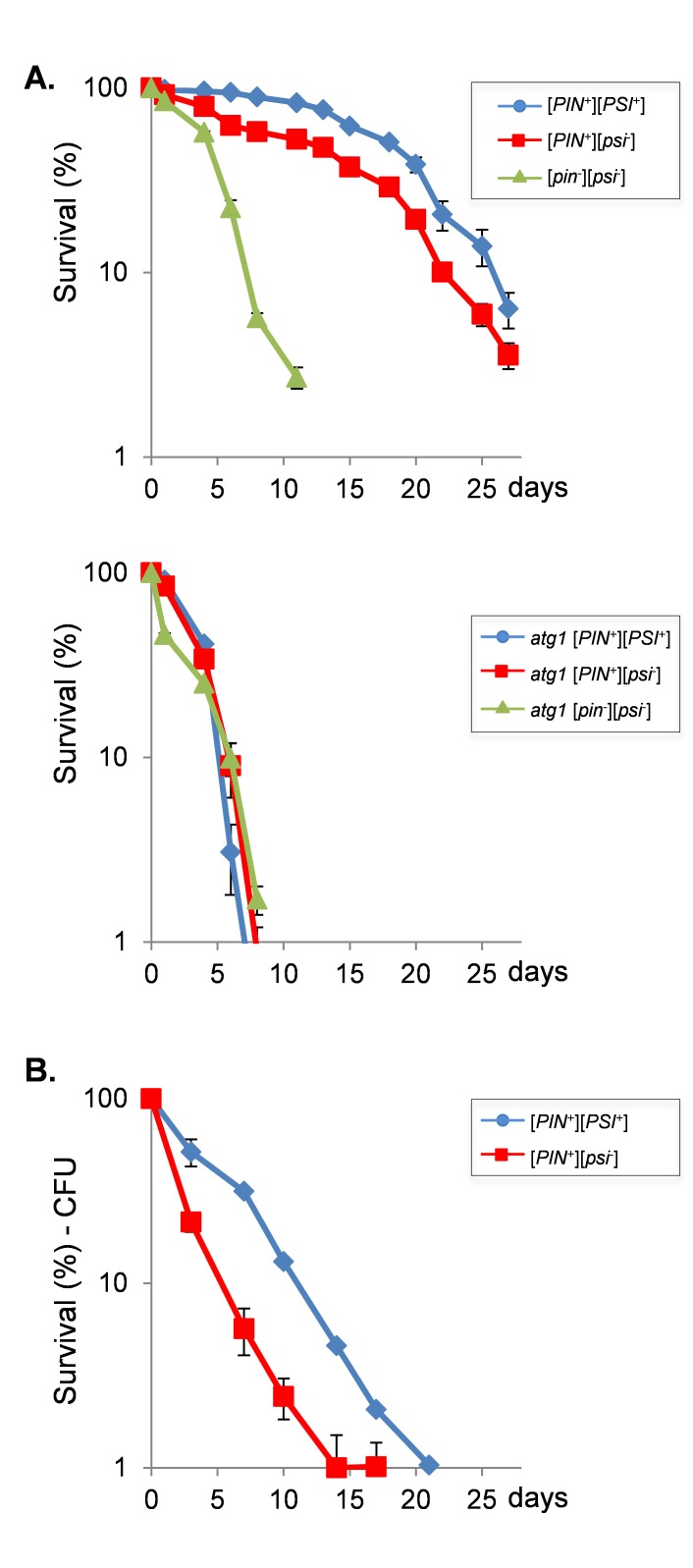
FIGURE 2: Prions improve chronological lifespan. **(A) **Chronological lifespan analysis, as determined by propidium iodide uptake to assess yeast cell death, is shown for [*PIN*^+^][*PSI*^+^], [*PIN*^+^][*psi*^-^] and cured [*pin*^-^][*psi*^-^] versions of wild-type and *atg1 *mutant strains**. **Cells were grown in SCD media for 3 days to reach stationary phase and then aliquots taken every 2-3 days for flow cytometry analysis based on propidium iodide uptake by non-viable cells as assayed through flow cytometry. Data shown are the means of at least three independent biological repeat experiments expressed as the percentage of viable cells out of 10000 cells analyzed. Error bars denote standard deviation. **(B) **Viability measurements are shown for [*PIN*^+^][*PSI*^+^] and [*PIN*^+^][*psi*^-^] strains grown under the same conditions as for (A) above. Viability is expressed as a percentage of day zero.

We next examined cell survival to determine whether the [*PSI*^+^] prion status of cells influences longevity. For this analysis, flow cytometry was used to monitor propidium iodide uptake to assess yeast cell death 24. The [*PIN*^+^][*PSI*^+^] strain showed a modest increase in maximal lifespan compared with a [*PIN*^+^][*psi*^-^] strain (Fig. 2A). Additionally, cell death was lower in the [*PIN*^+^][*PSI*^+^] strain over the entire lifespan compared with the [*PIN*^+^][*psi*^-^] strain suggesting that the [*PSI*^+^] prion improves viability during ageing. To verify this difference in ageing between [*PIN*^+^][*PSI*^+^] and [*PIN*^+^][*psi*^-^] strains, viability was monitored using colony formation assays. Whilst lifespan measured using the colony forming assay was shorter compared with the propidium iodide uptake assay, it confirmed that the [*PSI*^+^] strain maintained viability longer and had an increased lifespan compared with the [*psi*^-^] strain (Fig. 2B).

Treating cells with GdnHCl blocks the propagation of most yeast prions by inhibiting the key ATPase activity of Hsp104, a molecular chaperone that is absolutely required for yeast prion propagation 25,26. GdnHCl cures yeast cells of [*PSI*^+^] and all known prions. Curing the [*PIN*^+^][*PSI*^+^] strain with GdnHCl dramatically decreased maximal lifespan to 10 days suggesting that prions are beneficial during CLS (Fig. 2A). It should be noted however, that GdnHCl treatment can also potentially affect Hsp10-related processes which are unrelated to prions. Autophagy is known to be required for chronological longevity and for example loss of *ATG1 *reduces CLS 27. Similarly, we found that loss of *ATG1 *reduced CLS in the 74D-694 yeast strain used for our studies (Fig. 2A). Interestingly, longevity was comparable in the [*PIN*^+^][*PSI*^+^], [*PIN*^+^][*psi*^-^] and cured strains indicating that active autophagy is required for prion-dependent effects on longevity.

### [*PSI^+^*] cells have an increased rate of autophagy and decreased concentrations of amorphous protein aggregates

**Figure 3:**
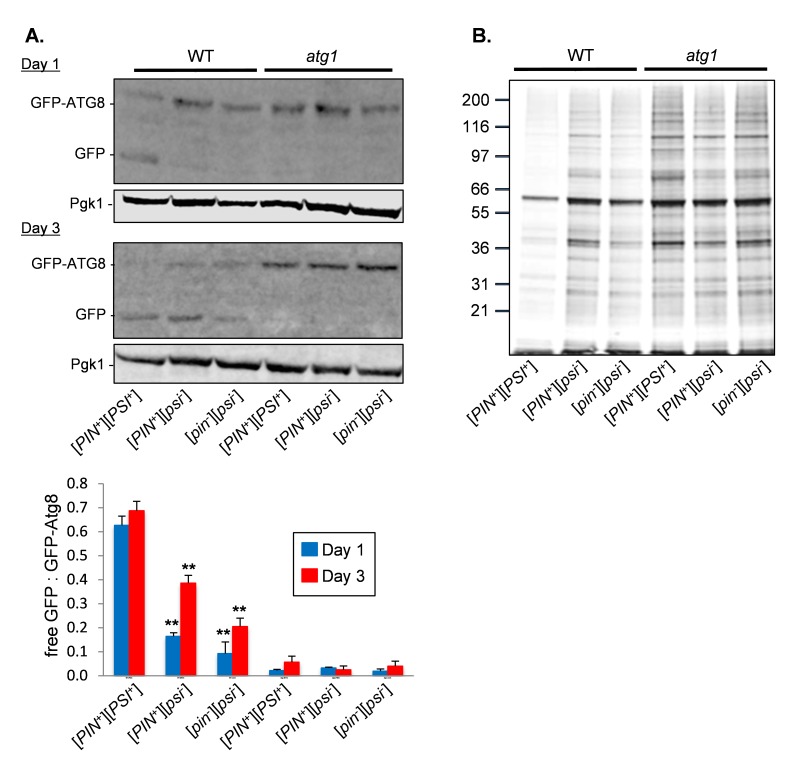
FIGURE 3: Increased autophagic flux in [*PSI^+^*] strains. **(A)** Autophagic flux was monitored in in the indicated strains expressing GFP-Atg8 during late exponential (day 1) and stationary phase growth (day 3). Increased free GFP is detected in the [*PIN*^+^][*PSI*^+^] strain indicative of autophagic flux. No free GFP is detected in the *atg1 *mutant strains which cannot initiate autophagy. Representative images are shown from triplicate experiments with quantification comparing the ratios of free GFP with GFP-Atg8 below. Significance is shown comparing the [*PIN*^+^][*PSI*^+^] versions of the wild-type and *atg1 *mutant strains with their corresponding [*PIN*^+^][*psi*^-^] and cured [*pin*^-^][*psi*^-^] versions. Statistical analysis was performed by one-way ANOVA: *p < 0.05, **p < 0.01. **(B)** Protein aggregates were isolated from the same strains as shown in panel A at day three of chronological growth and analyzed by SDS-PAGE and silver staining.

Given that the [*PSI^+^*]-prion status of cells affects CLS in an autophagy-dependent manner, we examined whether autophagy is altered in [*PSI^+^*] cells. We utilized a GFP-Atg8 fusion construct to follow the autophagy-dependent proteolytic liberation of GFP from GFP-Atg8, which is indicative of autophagic flux 28. In late exponential phase cells (day 1), more free GFP was detected in the [*PIN*^+^][*PSI*^+^] strain compared with the [*PIN*^+^][*psi*^-^] and cured [*pin*^-^][*psi*^-^] strains indicative of increased autophagic flux (Fig. 3A). By day three, there was also an increase in autophagic flux in the [*PIN*^+^][*psi*^-^] and cured [*pin*^-^][*psi*^-^] strains detected as an increase in free GFP. As expected, no autophagic activity was observed for the *atg1 *knockout mutants. This suggests that cells carrying the [*PSI^+^*] prion have increased autophagic activity which may be beneficial during CLS.

We next examined whether the increased autophagic activity in the [*PIN*^+^][*PSI*^+^] strain affects amorphous protein aggregation, which seemed likely given the previous studies which have suggested that autophagy plays a role in the clearance of misfolded and aggregated proteins 16,17. For this analysis, we used a biochemical approach where we grew cells to stationary phase (day 3) and isolated aggregated proteins by differential centrifugation, and removed any contaminating membrane proteins using detergent washes 29. The levels of protein aggregation were decreased in the [*PIN*^+^][*PSI*^+^] strain compared with the [*PIN*^+^][*psi*^-^] and cured [*pin*^-^][*psi*^-^] strains (Fig. 3B). This reduction in protein aggregation required autophagy since the levels of protein aggregation were comparable in the *atg1-*mutant version strains.

## DISCUSSION 

The majority of prion disease cases in humans occur in a sporadic manner, predominantly manifesting during later stages of life 8. Similarly, we found an age-dependent increase in the frequency of *de novo *[*PSI^+^*] formation during yeast chronological ageing, with the frequency of spontaneous formation increased approximately 40-fold in aged cells. Yeast has emerged as a powerful model to investigate the stochasticity of the ageing process and its contributing factors. The yeast CLS model more closely resembles the ageing of non-dividing cells such as neurons 18. Neuronal cells are post-mitotic in nature and rely on proteostasis mechanisms such as autophagy to facilitate the elimination of superfluous and damaged material. The age-dependent increase in the *de novo *formation of [*PSI^+^*] was exacerbated in an *atg1 *mutant suggesting that autophagy normally acts to suppress spontaneous prion formation during chronological ageing. Similarly, loss of autophagy has been found to cause neurodegenerative diseases in mice 16,17, supporting a protective role for autophagy in defending against age-associated abnormal protein accumulation and aggregation.

We found that that the presence of the [*PSI*^+^] prion confers a beneficial advantage during yeast chronological ageing, which correlates with enhanced autophagic flux. Given that the [*PSI*^+^]-status of cells improves viability during ageing, this may result in selection for [*PSI*^+^] in aged cells. Our data indicate that the presence of the [*PSI*^+^] prion acts to simulate autophagy which results in improved viability during ageing. There is previous evidence to suggest that the increased formation and accumulation of protein aggregates may exert a stimulatory effect on the autophagy pathway. For example, there is a correlation between the accumulation of PrP^SC^ and the enhanced activity of quality control pathways including endoplasmic reticulum chaperones, the unfolded protein response and autophagy 30. In agreement with the idea that enhanced autophagy aids protein homeostasis during ageing, we found reduced levels of amorphous protein aggregation in a [*PSI*^+^] strain, suggesting that autophagy provides a beneficial effect during chronological ageing by removing potentially harmful protein aggregates, including both amorphous and amyloid forms. Increasing autophagic flux would also presumably act to prevent further amyloid aggregation, potentially protecting against any negative impact of [*PSI*^+^] aggregation altering translation termination efficiency.

The [*PSI*^+^] prion causes a loss of function phenotype where translation termination activity is reduced due to the aggregation of the normally soluble Sup35 protein 10. The shift to the [*PSI*^+^] prion is thought to allow cells to reprogram gene expression such that new genetic traits become uncovered which may aid survival during altered conditions 31,32,33. However, as well as providing a selective advantage through altered gene expression, our data indicate that the [*PSI*^+^] prion can improve viability during ageing via modulation of autophagic flux. It is unclear what triggers the increased frequency of [*PSI*^+^] prion formation during ageing. One possibility is oxidative stress, since ROS-induced protein aggregation and mitochondrial dysfunction is a common feature in age-related diseases 34,35. In addition, ROS and oxidative stress are known to induce yeast and mammalian prion formation 36 which may account for increased [*PSI*^+^] formation observed during chronological ageing. Further research will be required to elucidate the exact signaling pathways and the range of quality control mechanisms that may be modulated through the direct and indirect action of the [*PSI*^+^] prion during yeast ageing.

## MATERIALS AND METHODS

### Yeast Strains

[*PIN^+^*][*PSI*^+^], [*PIN^+^*][*psi*^−^] and [*pin^-^*][*psi*^−^] derivatives of the wild-type yeast strain 74D-694 (*MATa ade1-14 ura3-52 leu2-3,112 trp1-289 his3-200)* were used for all experiments. The strain deleted for *ATG1 *(*atg1::*HIS3) has been described previously 12.

### Growth conditions

Yeast strains were grown at 30°C, 180 rpm in minimal SCD medium (2% w/v glucose, 0.17% yeast nitrogen base without amino acids, supplemented with Kaiser amino acid mixes, Formedium, Hunstanton, England). Chronological life span experiments were performed in liquid SCD media supplemented with a four-fold excess of uracil, leucine, tryptophan, adenine and histidine to avoid any possible artefacts arising from the auxotrophic deficiencies of the strains. Strains were cured by five rounds of growth on YEPD agar plates containing 4 mM GdnHCl.

### *De novo *[*PSI^+^*] formation

[*PSI*^+^] prion formation was scored by growth in the absence of adenine as described previously 12. [*PSI*^+^] formation was calculated based on the mean of at least three independent biological repeat experiments.

### Yeast Chronological Life Span Determination

CLS experiments were performed according to 37. Briefly, cells were cultured in liquid SCD media for 3 days to reach stationary phase and then aliquots taken every 2-3 days for flow cytometry analysis. 50 µl of 4 mM of propidium iodide (P.I.) was added to 950 µl of culture and cell viability was measured based on propidium iodide uptake by non-viable cells as assayed through flow cytometry. Flow cytometry readings were performed using a Becton Dickinson (BD) LSRFortessa™ cell analyser, BD FACSDiva 8.0.1 software) after staining with propidium iodide. For the colony forming assay, cultures were serially diluted and plated onto YEPD plates. Viable counts were recorded following three days growth and were expressed as a percentage of the starting viability.

### Protein analysis

Protein extracts were electrophoresed under reducing conditions on SDS-PAGE minigels and electroblotted onto PVDF membrane (Amersham Pharmacia Biotech). Bound antibody was visualised using WesternSure® Chemiluminescent Reagents (LI-COR) and a C-DiGit® Blot Scanner (LI-COR). Insoluble protein aggregates were isolated as previously described 38,39, with the following minor adjustments 29. Cell breakage was achieved by sonication (Sonifier 150, Branson; 8 x 5 s, Level 4) and samples were adjusted to equal protein concentrations before isolation of protein aggregates. Insoluble fractions were resuspended in detergent washes through sonication (4 x 5 s, Level 4). Insoluble fractions were resuspended in reduced protein loading buffer, separated by reducing SDS/PAGE (12% gels) and visualized by silver staining with the Bio-Rad silver stain plus kit. The induction of autophagy was confirmed by examining the release of free GFP due to the proteolytic cleavage of GFP-Atg8 28.
